# Antagonism between DNA and H3K27 Methylation at the Imprinted *Rasgrf1* Locus

**DOI:** 10.1371/journal.pgen.1000145

**Published:** 2008-08-01

**Authors:** Anders M. Lindroth, Yoon Jung Park, Chelsea M. McLean, Gregoriy A. Dokshin, Jenna M. Persson, Herry Herman, Diego Pasini, Xavier Miró, Mary E. Donohoe, Jeannie T. Lee, Kristian Helin, Paul D. Soloway

**Affiliations:** 1 Division of Nutritional Sciences, College of Agriculture and Life Sciences, Cornell University, Ithaca, New York, United States of America; 2 Department of Orthopaedic Surgery, School of Medicine, Padjadjaran State University–Hasan Sadikin General Hospital, Bandung, West Java, Indonesia; 3 Biotech Research and Innovation Centre, University of Copenhagen, Copenhagen, Denmark; 4 Centre for Epigenetics, University of Copenhagen, Copenhagen, Denmark; 5 Department of Molecular Cell Biology, Max-Planck-Institute of Biophysical Chemistry, Göttingen, Germany; 6 Massachusetts General Hospital, Boston, Massachusetts, United States of America; Netherlands Cancer Institute, Netherlands

## Abstract

At the imprinted *Rasgrf1* locus in mouse, a *cis*-acting sequence controls DNA methylation at a differentially methylated domain (DMD). While characterizing epigenetic marks over the DMD, we observed that DNA and H3K27 trimethylation are mutually exclusive, with DNA and H3K27 methylation limited to the paternal and maternal sequences, respectively. The mutual exclusion arises because one mark prevents placement of the other. We demonstrated this in five ways: using 5-azacytidine treatments and mutations at the endogenous locus that disrupt DNA methylation; using a transgenic model in which the maternal DMD inappropriately acquired DNA methylation; and by analyzing materials from cells and embryos lacking SUZ12 and YY1. SUZ12 is part of the PRC2 complex, which is needed for placing H3K27me3, and YY1 recruits PRC2 to sites of action. Results from each experimental system consistently demonstrated antagonism between H3K27me3 and DNA methylation. When DNA methylation was lost, H3K27me3 encroached into sites where it had not been before; inappropriate acquisition of DNA methylation excluded normal placement of H3K27me3, and loss of factors needed for H3K27 methylation enabled DNA methylation to appear where it had been excluded. These data reveal the previously unknown antagonism between H3K27 and DNA methylation and identify a means by which epigenetic states may change during disease and development.

## Author Summary

Methylation of DNA and histones exert profound and inherited effects on gene expression. These occur without changes to the underlying DNA sequence and are considered epigenetic effects. Disrupting epigenetic states can cause developmental abnormalities and cancer. Very little is known about how locations in the mammalian genome are chosen to receive these chemical modifications, or how their placement is regulated. We have identified a DNA sequence that acts as a methylation programmer at the *Rasgrf1* locus in mice. It is required for methylation of nearby DNA sequences and can also influence the levels of local histone methylation. The methylation programmer has different effects on paternally and maternally derived chromosomes, directing DNA methylation on the paternal allele and histone H3 lysine 27 trimethylation on the maternal allele. These two methylation marks are not only mutually exclusive; they are also mutually antagonizing, whereby one blocks the placement of the other. Manipulations that cause aberrant changes in the levels of one of these marks had the opposite effect on the other mark. These observations identify novel mechanisms that specify epigenetic states *in vivo* and provide a framework for understanding how pathological epigenetic changes can arise, including those emerging at tumor suppressors during carcinogenesis.

## Introduction

In mammals, imprinted loci are expressed from only one allele. Accompanying and controlling monoallelic expression are allele-specific epigenetic modifications influenced by an imprinting control region (ICR). Within this region, there is a differentially methylated domain (DMD) that is subject to acquisition of epigenetic modifications, typically DNA methylation and histone modifications. These modifications are placed in a parent-of-origin specific manner and impose an epigenetic state that dictates allele-specific gene expression at imprinted loci [1].

Previously, we characterized the mechanisms by which the ICR controls allele-specific methylation and expression at the imprinted *Rasgrf1* locus. The ICR, located 30 kbp upstream of the transcriptional start site, is a binary switch consisting of a repeated element and the DMD. The repeated element functions as a methylation programmer, that is necessary for the establishment and maintenance of DNA methylation at the DMD on the paternal allele and sufficient for establishing gametic imprints in both germlines ([2],[3] and YJP, HH, AML, Ying Gao and PDS, in preparation). The DMD is a methylation sensitive enhancer blocker that binds CTCF on the unmethylated maternal allele and limits enhancer to promoter interactions, silencing the maternal allele [4]. DNA methylation that is directed to the paternal DMD by the repeats prevents CTCF binding, allowing expression of the paternal allele. The repeats constitute the first identified, and one of only a few known, naturally occurring DNA methylation programmers in mammals [5]–[8].

Epigenetic analysis of *Rasgrf1* done by others examined DNA methylation across an expanded region centered on the ICR ([9] and Hisato Kobayashi and Hiroyuki Sasaki unpublished data) and histone modifications at the ICR [10]. The DNA methylation data suggested that a broader DMD exists in somatic tissue and in the male germline than was previously appreciated [9]. The histone methylation data indicated that several allele-specific histone modifications accompany the DNA methylation differences, including H3K27me3 and H4K20me3 on the maternal allele and H3K9me3 on the paternal allele [10].

The *Rasgrf1* locus presents some unusual paradoxes: The paternal allele is active yet it carries DNA methylation and other repressive marks, whereas the maternal allele is silent and lacks DNA methylation but carries other repressive marks. It is unclear if and how the primary DNA sequence controls each of these parent-specific marks. We have identified the DNA sequences that are necessary and sufficient for programming the establishment and maintenance of DNA methylation on the paternal allele, however, nothing is known about the *cis*-acting DNA sequences that control placement of repressive histone modifications in this region, or whether there is any coordination between the histone and DNA methylation modifications. In many organisms, distinct epigenetic marks coordinately determine the transcriptional status of genes. For instance, recruitment of DNA methylation can depend upon pre-established histone H3 methylation at lysine 9 [11]–[13]; histone modifications can be lost when DNA methylation is impaired [14]; and some histone modifications become redistributed in histone methyltransferase mutants [15].

Here we report the analysis of a 12 kbp region at *Rasgrf1* for locations bearing histone modifications and DNA methylation. Our data reveal the mutual exclusion of the repressive H3K27 methylation and DNA methylation modifications. Furthermore, by experimentally manipulating the levels of DNA and H3K27 methylation possible at the locus, we demonstrate that these two marks are mutually antagonistic, whereby the placement of one mark prevents the placement of the other, and removal of one mark allows the encroachment of the other. Additionally, we found that the tandem repeat sequences, which are necessary and sufficient for programming DNA methylation marks, are also important for directing H3K27 and H3K9 modifications to the proximal DMD and that H3K9 methylation is needed for optimum establishment of DNA methylation on the paternal allele.

## Results

### The *Rasgrf1* Repeats Program DNA Methylation Only at the 400 nt DMD in the Male Germline

**Figure 1:**
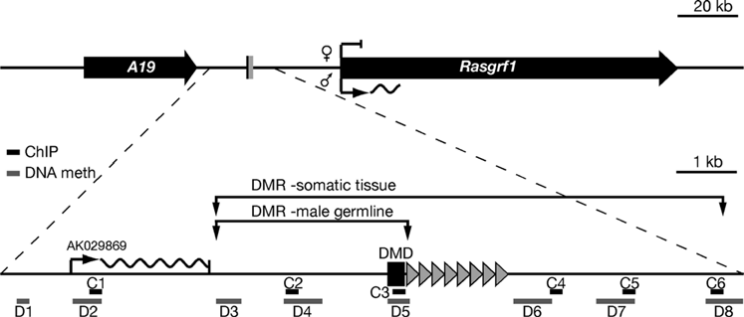
Schematic view of the imprinted *Rasgrf1* locus. A 220 kbp region (top) contains the paternally-expressed *A19* and *Rasgrf1* transcripts (black, rightward pointing arrows) and the ICR (black rectangle). Detailed view of 12 kbp centered on the ICR (bottom) includes the originally defined 400 nt core differentially methylated domain (DMD) and tandem repeats (triangles), which constitute the DNA methylation programmer. Amplicons for ChIP (C1–C6) and DNA bisulfite sequencing (D1–D8) are indicated. The sites of the germline and somatic DMR as described by Kobayashi *et al.* are shown ([9] and Hisato Kobayashi and Hiroyuki Sasaki, unpublished).

There are two regions rich in C and G residues and CpG dinucleotides over a 200 kbp interval at the *Rasgrf1* locus. One CpG cluster is in the ICR and the other in the promoter region of *Rasgrf1* (Figure S1A, B, C). By analyzing the DNA methylation pattern of these two CpG clusters in somatic DNA using methylation sensitive restriction enzymes, we found that only the DMD CpG cluster is methylated while the one in the promoter is not (Figure S1D). When Kobayashi *et al.* performed a comprehensive analysis of allele-specific DNA methylation at the *Rasgrf1* ICR in embryonic day 12.5 DNA and in the male germline, they observed that the somatic and germline DMD was larger than had been previously appreciated ([9] and Hisato Kobayashi and Hiroyuki Sasaki unpublished data). We expanded upon this by characterizing the distribution of both histone modifications and DNA methylation over a 12 kbp region centered on the DMD within the ICR, and also by evaluating the influence of the tandem repeats within the ICR on these epigenetic marks (Figure 1).

**Figure 2:**
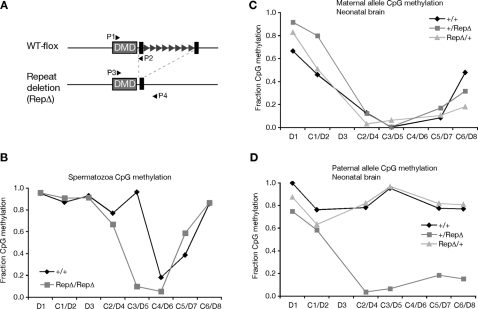
Distribution of DNA methylation over 12 kbp spanning the ICR. (A) Alleles analyzed. The functionally wild type (WT-flox) allele has a loxP sites (black rectangles) flanking the tandem repeat element (triangles) in the ICR and behaves like an unmodified wild type (+) allele [3]. Cre recombinase was used to delete the repeat element (RepΔ). Primers P1–P2 amplify the C3 region from + or WT-flox alleles; P3 and P4 amplify the RepΔ allele. (B) CpG methylation in spermatozoa DNA isolated from wild type (+/+) mice or mice homozygous for the repeat element deletion (RepΔRepΔ). (*C*) Maternal allele CpG methylation in neonatal brain of mice that harbor two wild type (+/+) alleles, a paternal repeat element deletion (+/RepΔ) or maternal repeat element deletion (RepΔ/+). Maternal allele is shown first. (D) Paternal allele CpG methylation of DNAs from (C). The fraction of CpGs methylated in each segment (D1 through D8) is reported, calculated as the fraction of CpGs methylated in all clones we sequenced from bisulfite treated DNAs. A total of 500 templates were sequenced. Table S2 reports the number of sequences for the two parental alleles in each of the regions assayed (D1–D8) in the various somatic and germline DNAs.

We performed bisulfite sequencing to characterize DNA methylation in 86 CpGs present in eight segments (labeled D1 through D8 in Figure 1) containing 4,118 bp from the 12,020 bp interval. Our analysis of methylation in the soma used DNA from neonatal brain and our analysis in the male germline used DNA isolated from sperm. Somatic DNAs were from F1 progeny of 129S4Jae and PWK strains. Polymorphisms between these strains allowed us to determine which bisulfite sequences were from the maternal and paternal alleles in the soma. The 129S4Jae-derived allele was either wild type or lacked the *Rasgrf1* tandem repeats constituting the DNA methylation programmer (Figure 2A). Sperm DNAs were from mice homozygous for wild type or tandem repeat-deficient alleles of *Rasgrf1*. Our characterization of the somatic methylation states from animals carrying the wild type 129S4Jae allele was in strong agreement with the results of Kobayashi, even though sources of somatic DNAs differed: Kobayashi used midgestation embryos. In neonatal brain DNA, we detected paternal allele-specific DNA methylation, which covers at least the 7.6 kbp interval between segments D4 through D7 and includes the ICR. We also found a region of methylation on both alleles over a 1.4 kbp interval upstream of the ICR containing segments D1 and D2. None of the somatic DNA methylation patterns changed on either the paternal or maternal alleles in mice harboring a deletion of the tandem repeats on the maternal allele (Figure 2C, D). In contrast, all paternal allele-specific DNA methylation we detected in regions D4 to D8 was lost from somatic DNA when the tandem repeats were absent from the paternal allele (Figure 2D). This indicates that the range of action of the *Rasgrf1* DNA methylation programmer within the tandem repeats is not confined to the narrowly defined 400 nt DMD previously studied, but its reach spans at least 7 kbp in somatic tissue.

Imprinted DNA methylation patterns that are established in the germlines are typically maintained and can even spread during somatic development. To determine the extent of the methylation in sperm DNA and the range of action of the DNA methylation programmer in the male germline, we performed bisulfite analysis on sperm DNA from mice carrying an intact repeat element and also from mice carrying a deletion of the repeats. In mice with the intact repeats, we found that *Rasgrf1* methylation in sperm DNA was present not only the originally defined 400 bp DMD, but it extended an additional 1.6 kbp upstream, in agreement with results from Hisato Kobayashi and Hiroyuki Sasaki (unpublished). However, in mice bearing a deletion of the repeats that constitute the *Rasgrf1* DNA methylation programmer, only the DNA methylation at the originally defined DMD was lost. The DNA methylation on the additional 1.6 kbp was unaffected, indicating that the range of action of the *Rasgrf1* DNA methylation programmer in the tandem repeats is limited to the 400 bp proximal DMD in the male germline (Figure 2B). Because loss of DNA methylation on that narrowly defined sequence was sufficient to disrupt imprinted expression of *Rasgrf1* [2], we infer that this differentially methylated portion of the locus is essential for its imprinting and we refer to it as the core DMD.

### Allele-Specific Histone Modifications Are Present at the DMD and Depend on the DNA Methylation Programmer

We next characterized histone methylation status across the same 12 kbp interval over which the DNA methylation was characterized. Specifically, we sought to determine where histone modifications were distributed, if any modifications were allele-specific, if their placement required the same DNA methylation programmer that imprinted DNA methylation requires, and if there is any coordination between modification states on histones and DNA. We limited our analysis to di-, and tri-methylation of histone H3 at lysine 9 and 27 because they are associated with gene silencing and DNA methylation, which are observed at the maternal and paternal alleles respectively. For this analysis, we performed chromatin immunoprecipitation (ChIP) using mouse embryonic fibroblasts (MEFs) and antibodies specific to H3K9me2, H3K9me3, H3K27me2, and H3K27me3. Our initial tests were controls to verify that the antibodies detected histone modifications with proper specificity (Figure S2). For these tests, we amplified immunopreciptates using primers from *Charlie*, *Actin* and *Hoxa9*. H3K9me2 and H3K27me2 are known to reside at *Charlie* [16], H3K9me3 at *Actin*, and H3K27me3 at *Hoxa9* [17]. The expected PCR products were observed for each immunoprecipitation, indicating the antibodies were indeed specific. In addition, PCRs done using DMD primers detected only H3K9me3 and H3K27me3 at the DMD; therefore, subsequent ChIP studies primarily used antibodies recognizing these marks (Figure S2).

**Figure 3:**
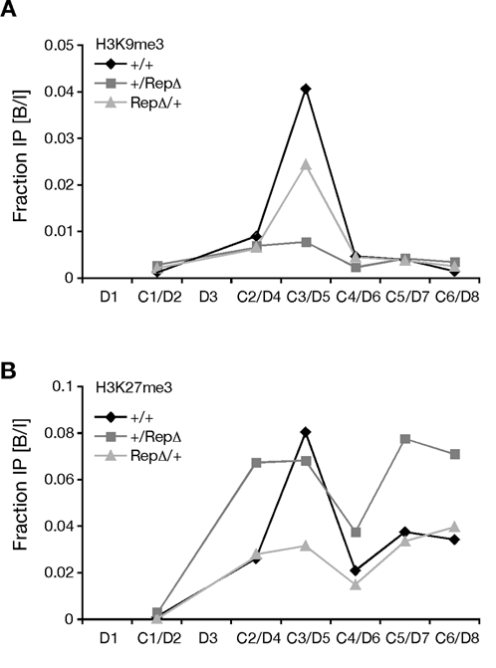
Distribution of methylated histones at *Rasgrf1.* (A) H3K9me3 and (B) H3K27me3 distribution over 12 kbp spanning the ICR in MEFs isolated from wild type (+/+) mice and mice with a deletion of the tandem repeat element from the paternal (+/RepΔ) or maternal (RepΔ/+) alleles. Data plotted as fraction bound over input, as measured by quantitative real-time PCR (Q-PCR). Note that in +/RepΔ MEFs, which had lost DNA methylation, the H3K27me3 mark encroached into sites where it was originally absent in +/+ MEFs.

We then extended our H3K27me3 and H3K9me3 analysis to six segments (labeled C1 through C6 in Figure 1) that included 10,451 bp surrounding the core DMD and methylation programmer using two separate immunopreciptates from wild type MEFs, and MEFs carrying a deletion of the DNA methylation controlling repeats (RepΔ. Because we used two immunoprecipitates, these analyses report the general distribution of histone marks in the region rather than providing reliable quantification of their abundance. Our PCRs in regions C1, C2, C4, C5 and C6 did not distinguish the parental alleles and our PCR of the DMD at C3 used wild type allele-specific primers. The ChIP analysis of wild type MEFs indicated that both H3K9me3 and H3K27me3 were most abundant at the core DMD at region C3 with some H3K27me3 signal extending downstream of the tandem repeats (Figure 3). Analysis of MEFs carrying a deletion of the DNA methylation controlling repeats suggested that the repeats could influence the distribution of histone modifications at the DMD and elsewhere in the region.

**Figure 4:**
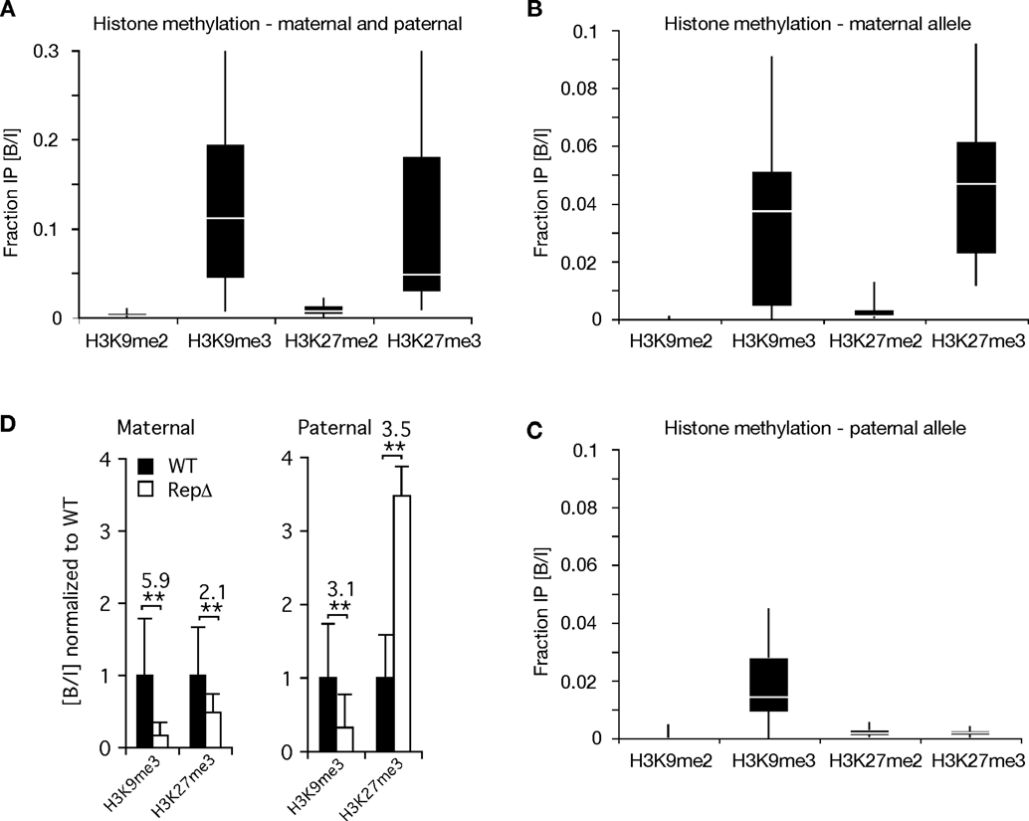
Allele specific histone modifications on the *Rasgrf1* core DMD and their sensitivity to methylation programming repeats. (A–D) Quantitative PCR analysis of immunoprecipitates and unprecipitated input materials was used to calculate the fraction of input material precipitated by each antibody (reported as Bound/Input, B/I). Box plots report distribution of B/I values from 6–12 individual precipitations, each analyzed in triplicate. (A) Analysis of the DMD using wild type cells, which does not distinguish the alleles, shows the DMD is enriched for H3K9me3 and H3K27me3 (one-way ANOVA and multiple comparison: Hsu's MCB, a = 0.05). (B) Maternal allele specific analysis of the wild type DMD using wild type allele-specific primers and immunoprecipitates from heterozygous +/WT-flox or +/RepΔ cells. Modified alleles are shown in Figure 2A. (C) Paternal allele specific analysis of the wild type DMD performed as in *B* but using WT-flox /+ or RepΔ/+ cells. In *B* and *C*, results did not depend on which heterozygote was used indicating that no interactions between the alleles affected the histone modifications analyzed. H3K9me3 is present on both alleles, while H3K27me3 is present only on the maternal allele (p<0.01). (D) Sensitivity of H3K9me3 and H3K27me3 methylation to the tandem repeats. Graph represents B/I values from ChIP analysis of the RepΔ allele, obtained using primers P3/P4 in Figure 2A (white bars), normalized to measurements from the wild type (WT) allele, obtained using primers P1/P2 in Figure 2A (black bars). Analyses used RepΔ/+ and +/RepΔ cells to assess the importance of the maternal and paternal repeats respectively. Deletion of the maternal repeats caused significant decreases in H3K9me3 and H3K27me3 on the maternal allele (**, p<0.01). Deletion of the paternal repeats caused a significant decrease in paternal H3K9me3 (**, p<0.01) and an increase in paternal H3K27me3 (**, p<0.01). Fold increase or decrease is indicated above each pair of bars. Deletions of the repeat on one allele did not affect the histone states on the homologous allele (not shown). P values determined by a mixed model with log-transformed B/I values, a fixed independent variable for allele (wild type or repeat deletion) and a random variable IP.

To provide statistically significant measures of methylated H3K9 and H3K27 at the DMD and to assess if any modifications were allele-specific, we analyzed a total of six to twelve independent immunoprecipitations by quantitative PCR using primers spanning the DMD at region C3 (Figure 2A). Our data confirmed that the DMD is enriched for trimethylated lysines but lacks dimethylated ones (Figure 4A). To determine if these histone marks over the DMD were on the maternal or paternal alleles, we repeated the ChIP assays using mice carrying the engineered polymorphisms shown in Figure 2A that enabled us to amplify the wild type maternal and paternal DMD sequences separately. Results demonstrated that the maternal allele has H3K9me3 and H3K27me3, whereas the paternal DMD has only the H3K9me3 mark (Figure 4B and C). This is in partial agreement with other data describing H3K27me3 as being maternal allele specific and H3K9me3 as being paternal allele specific at *Rasgrf1* [10]. H3K9me3 that we detect on the two alleles may be placed by different mechanisms. Our data correlate well with previous findings that DNA methylation can be coregulated with H3K9me3 [11],[13],[18],[19], but generally not with H3K27me3 [20],[21].

Because the tandem repeats act as a DNA methylation programmer, playing an essential role both in establishment and maintenance of DNA methylation at the DMD ([2],[3] and YJP, HH, AML, Ying Gao and PDS, in preparation), we wanted to determine if they also influence placement of methylated histone marks at the DMD. We did this by repeating the ChIP analysis using MEFs carrying a deletion of the repeats (RepΔ) and amplifying the wild type allele and the mutated allele separately. Our analysis showed that the repeat element indeed has a significant influence of histone modification status at the DMD, in addition to controlling its DNA methylation (Figure 4D): When the repeats were absent from the maternal allele, the levels of maternal allele-specific H3K27me3 and H3K9me3 were respectively 1/2 and 1/6^th^ the levels seen when the repeats were present. Similarly, when the repeats were absent from the paternal allele, the level of paternal allele-specific H3K9me3 was 1/3^rd^ that seen when the methylation programmer was absent. Interestingly, deletion of the repeats from the paternal allele led to a three-fold increase in the accumulation of H3K27me3 on the paternal allele. This is consistent with our locus wide ChIP analysis spanning intervals C1 to C6, which suggested H3K27me3 can encroach into areas where it is normally absent, both 5′ and 3′ of the DMD, when the paternal repeats are deleted (see sites C2, C5, C6 in Figure 3B). These observations provide evidence that DNA methylation and H3K27me3 are mutually exclusive epigenetic marks at *Rasgrf1*. When we superimposed the DNA and H3K27 methylation data for wild type animals and animals carrying a deletion of the paternal methylation programmer from Figures 2 and 3, the mutual exclusion of H3K27me3 and DNA methylation over the core DMD was apparent (shown separately in Supporting Figure S3A and B for clarity).

### Antagonism between H3K27 and DNA Methylation

Mutual exclusion of H3K27me3 and DNA methylation can arise by different mechanisms. In one scenario, the two marks may be placed in different compartments of the nucleus and the DNA cannot reside in both places. Alternatively, distinct factors needed for H3K27me3 and DNA methylation may require the same DNA binding site, which cannot be simultaneously occupied by the two sets of factors. A third possibility is that DNA and H3K27 methylation are mutually antagonizing, whereby one inhibits placement of the other. This last possibility is mechanistically different from mere mutual exclusion. If antagonism between these two marks is occurring, then we can predict what happens to one mark if the other is experimentally manipulated.

**Figure 5:**
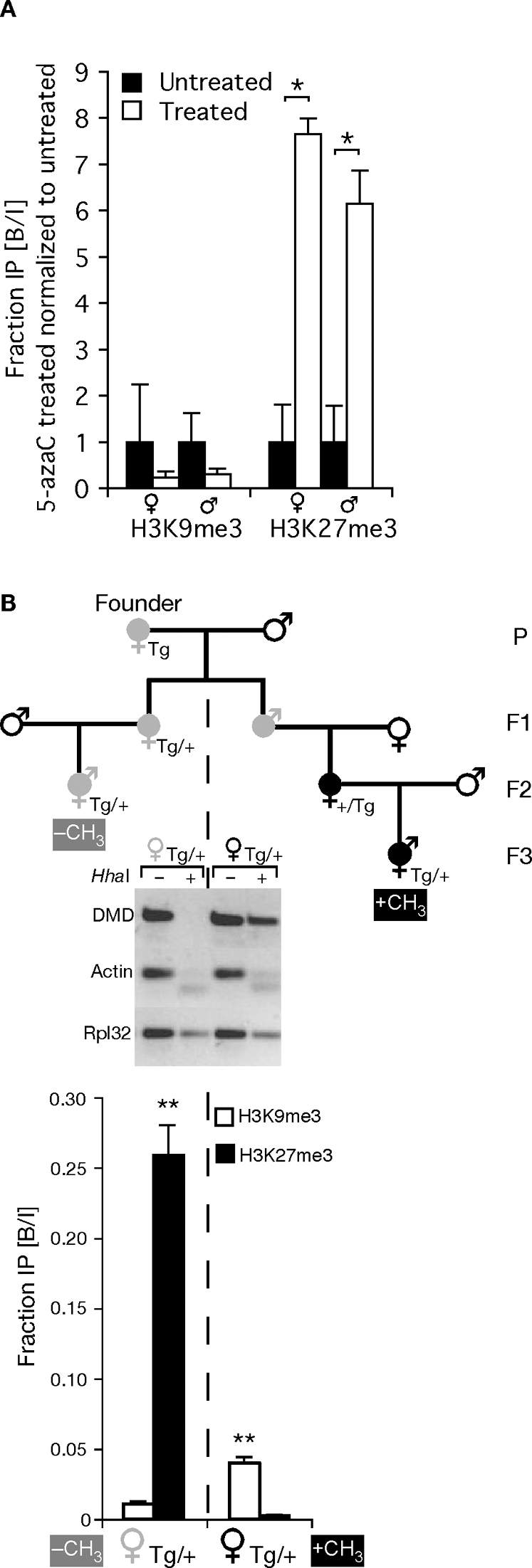
DNA methylation excludes H3K27me3 from the *Rasgrf1* ICR, while loss of DNA methylation lead to acquisition of H3K27me3. (A) Allele specific ChIP analyses using MEFs treated for 24 hours with 0.4 µM of 5-azacytidine (5-azaC), a DNA methyltransferase inhibitor. B/I values from treated cells were normalized to the values from untreated control cells. H3K27me3 was significantly increased on both alleles after 5-azaC treatment (* p<0.05). (B) Transgene specific ChIP analyses. Upper panel: Pedigree of transgenic mice from which MEFs were isolated. Transgene positive animals indicated by filled grey or black symbols. A female parental generation (P) transgenic founder and her F1 progeny have an unmethylated transgene (filled gray symbols). Strict maternal inheritance of the transgene preserved its unmethylated state (left portion of pedigree), whereas any intervening transmission by males caused methylation (filled black symbols) that persisted even after subsequent transgene transmission by females (right portion of pedigree). Middle panel: Methylation state at five *Hha*I sites in the transgene. DNAs were prepared from MEFs grown from the last progeny of both halves of the pedigree shown in *A* that carried a maternally transmitted transgene. DNAs were undigested (−) or digested with the methylation-sensitive enzyme *Hha*I (+) prior to PCR amplification using transgene specific primers. As a control for *Hha*I digestion, DNAs were amplified using primers from *Actin* that flank an unmethylated *Hha*I site. As a control for the PCR reaction, DNAs were amplified using primers from *Rpl32* that span an interval lacking *Hha*I sites. Lower panel: ChIP analysis for H3K9me3 and H3K27me3 using MEFs whose maternal transgenic DMD was methylated (black ♀ symbol, right two bars) or unmethylated (grey ♀ symbol, left two bars). There is a significant enrichment of H3K27me3 on the unmethylated maternal transgene and H3K9me3 on the methylated maternal transgene (**p<0.01). P values determined by a mixed model with log-transformed B/I values, a fixed independent variable for 5-azaC treatment (A) or parental transmission (B) and a random variable IP.

In order to explore more directly the possible antagonism between DNA methylation and H3K27me3, we repeated our allele-specific ChIP studies using MEFs that had been treated with the DNA methyltransferase inhibitor 5-azacytidine. If DNA methylation can antagonize H3K27 methylation, then we expected that 5-azacytidine treatments should increase the levels of H3K27me3 at the DMD as assayed by ChIP. This is precisely what we observed. 5-azacytidine treatments increased the signals from our ChIP analysis by more than six fold when we assayed H3K27me3 on the two parental alleles (Figure 5A). Although the maternal allele lacks imprinted DNA methylation, there is DNA methylation at sites D1, D2 and D8. Reductions in methylation at those sites might augment accumulation of H3K27me3 across the entire region.

If DNA methylation antagonizes H3K27 methylation, then an additional expectation is that inappropriate placement of DNA methylation on the maternal DMD should exclude accumulation of H3K27me3 marks. To test this, we took advantage of a transgenic system we developed to test if the tandem repeats, which are necessary for programming DNA methylation at *Rasgrf1*, are sufficient to impart imprinted methylation to the DMD at an ectopic location in the genome. Independent transgenic founders harboring three to five ectopic copies of the *Rasgrf1* ICR underwent proper establishment of DNA methylation at the transgenic DMD in the male germline and erasure of that methylation in the female germline, recapitulating the essential features of imprinted methylation establishment seen at the endogenous locus (YJP, HH, AML, Ying Gao and PDS in preparation). We were able to distinguish the transgenic ICR from the endogenous copy because the transgenic repeats were flanked with loxP sites and had the same structure as the WT-flox allele shown in Figure 2A. This allowed us to assay DNA methylation and perform ChIP analysis of the transgene. The transgene was useful for the studies we describe here because the unmethylated state that was established on the transgene after female transmission could not be maintained if there was any history in the pedigree of transmission through a male (Figure 5B). This system of transgenerational epigenetic memory allowed us to generate two different sets of MEFs, both of which were derived by maternal transmission of the transgene from a common founder. For one set of MEFs, the transgene was unmethylated at the transgenic DMD, whereas in the second set, the same transgene was methylated on the DMD (Figure 5B, upper two panels). If there is antagonism between H3K27me3 and DNA methylation at *Rasgrf1*, then we predicted our MEFs with a methylated transgene should exclude H3K27me3, whereas our MEFs with an unmethylated transgene should allow its placement. This is also precisely what we observed (Figure 5B, lower panel), providing additional independent evidence that DNA methylation antagonizes placement of H3K27me3.

We next wondered if the antagonism between DNA methylation and H3K27me3 might be reciprocal, meaning; H3K27me3 is able to exclude DNA methylation. To test this possibility we analyzed the DNA methylation state of the *Rasgrf1* DMD in ES cells, embryoid bodies or trophoblast outgrowths that lack either of two factors needed for H3K27me3 by the PRC2 complex. PCR2 includes SUZ12, EED and EZH2, the H3K27 methyltransferase. YY1, the mammalian ortholog of the *Drosophila* PHO protein, is a DNA binding factor that binds to EED and recruits PRC2 to sites of action [22]–[24]. Mice and cells with deficiencies in either SUZ12 or YY1 fail to acquire normal levels of H3K27me3 genome wide [22],[25],[26], and the deficiency is lethal for mice, but SUZ12-deficient ES cells are viable [26],[27].

**Figure 6:**
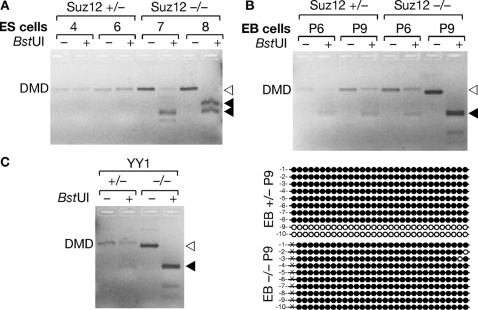
Loss of H3K27 methylation potential enables inappropriate DNA methylation. (A, B) DNA methylation analysis at *Rasgrf1* in *Suz12* +/− or −/− ES cells (A) and embryoid bodies (EB, *B*). EB were prepared from ES cells after 6 (P6) or 9 (P9) days in culture. Methylation analysis was by COBRA (A) and (B, top) and by bisulfite sequencing of P9 EB DNA (B, bottom). (C) COBRA analysis of trophoblast outgrowths from embryos lacking YY1, a CTCF and PRC2 co-factor. In COBRA, bisulfite treated DNAs were amplified using the core DMD spanning primer pair from D5 in Figure 1, and PCR products were left undigested (−) or digested with *Bst*UI (+) prior to electrophoresis. Six *Bst*UI sites are in the amplicon. DNA methylation preserves the sites in bisulfite treated DNA, whereas the sites are lost in unmethylated DNA. When one allele is fully methylated and the other unmethylated, COBRA will produce an equal quantity of uncut and cut bands after *Bst*UI digestion. When the maternal allele acquires DNA methylation, amounts of digested products will increase; when the paternal allele loses DNA methylation, fewer digested products appear. Open triangles, unmethylated DNA; filled triangles, methylated DNA.

If conditions necessary for proper placement of H3K27me3 are in fact required to antagonize placement of DNA methylation on the maternal DMD of *Rasgrf1*, then DNA methylation at the DMD will increase in the absence of SUZ12 and YY1. Because DNA methylation at the *Rasgrf1* DMD is normally restricted to the paternal allele, which is completely methylated, any increase in DNA methylation would arise on the maternal allele. To monitor the level of *Rasgrf1* DMD methylation in SUZ12- and YY1-deficient materials, we used COBRA [28]. This involved treating DNAs with bisulfite and amplifying them using primers not overlapping with CpG dinucleotides, which will amplify templates without bias for either methylation state. We then digested the PCR products with *BstUI*. Methylated templates will retain the *BstUI* recognition site (CGCG) after amplification and will be digested, whereas unmethylated templates that underwent bisulfite conversion of either CG in the recognition site to TG will resist digestion. There should be an equal amount of digested and undigested PCR product when the maternal allele is completely unmethylated and the paternal allele is completely methylated. This is what we saw in embryoid bodies and blastocysts that were heterozygous respectively for the *Suz12* and *Yy1* mutations. This indicated that our COBRA assays accurately reported the presence of both methylated and unmethylated templates expected in these *Suz12* and *Yy1* expressing materials; however, it is not clear why *Suz12* heterozygous ES cells did not show this pattern. When we performed COBRA analysis on SUZ12-deficient embryoid bodies (EB) that had differentiated for six (P6) or nine (P9) days *in vitro* (Figure 6A, B) or on trophoblast outgrowths from YY1-deficient blastocysts (Figure 6C), we found a dramatic increase in the levels of the digested PCR product in three out of four samples of *Suz12* −/− material and in the *Yy1* −/− material, indicating that loss of SUZ12 or YY1 resulted in increased *Rasgrf1* DMD methylation. The near complete acquisition of DNA methylation in P9 EB lacking SUZ12 was confirmed by bisulfite sequencing (Figure 6B lower panel), whereas unmethylated DNA was present in EB with a single functional allele of *Suz12*, though it is possible there is a quantitative increase in *Rasgrf1* DNA methylation when only one functional copy of *Suz12* is present. We do not know why only three out of four of the *Suz12* −/− DNAs show hypermethylation. This could be an artifact of cell cultures, which can exhibit frequent and cyclic changes in DNA methylation [29]. Also, mutation of *Eed*, another component of PRC2, is known to cause hypermethylation and hypomethylation simultaneously, depending upon which CpGs are queried [30]. Given these precedents, it is possible that the eight CpGs we assayed in the *BstUI* sites are predominantly hypermethylated in cultured cells lacking SUZ12. Nonetheless, our data provide evidence that the antagonism between DNA and H3K27 methylation is reciprocal and that H3K27me3 antagonizes placement of DNA methylation. Furthermore, this mutual antagonism exists in at least three DNA sources: MEFs, embryoid bodies and trophoblast outgrowths.

**Figure 7:**
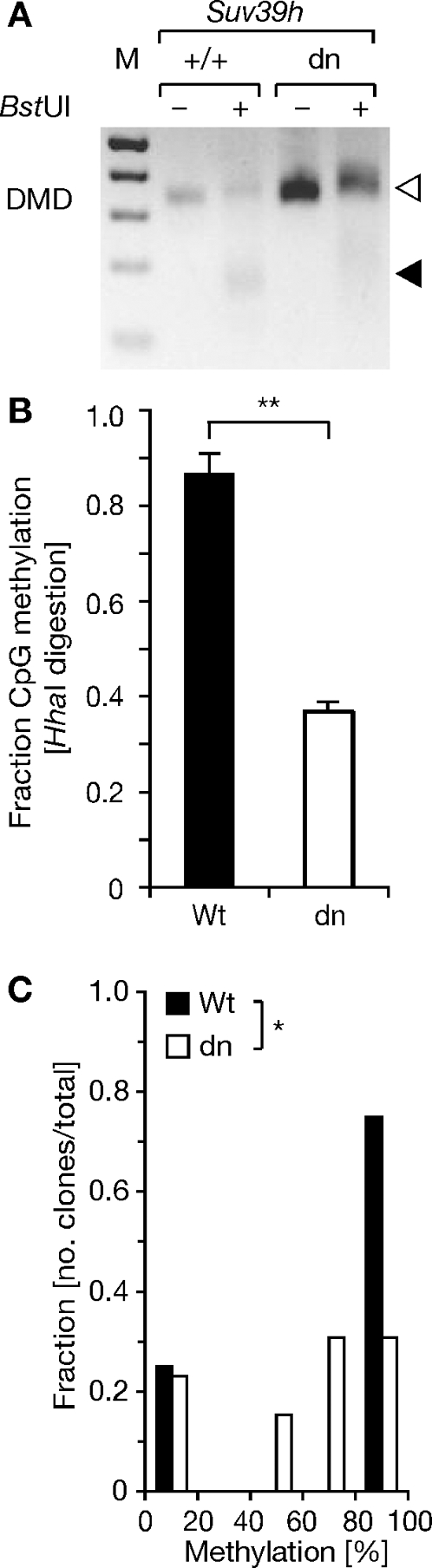
Loss of *Suv39h1* and *Suv39h2* causes reductions in *Rasgrf1* DNA methylation. (A) COBRA analysis of testes DNAs isolated from wild type (WT, +/+) mice or animals deficient for both *Suv39h1* and *Suv39h2* (dn), performed as described in Figure 6. (B) Methylation analysis using the PCR assay described in Figure 5B, middle panel, done in triplicate and analyzed by Q-PCR. A significant decrease of DNA methylation is observed in dn material (Student's t-test, p<0.01). (C) Bisulfite treated DNAs were amplified, cloned and sequenced. Data reported as the quintile distribution of methylation densities observed for the collection of clones (13 dn clones and 8 WT). The dn samples exhibit a significant excess of hypomethylated CpGs as assessed by likelihood ratio Chi-square analysis (p<0.05).

We also explored the relationship between H3K9 and DNA methylation at *Rasgrf1*. H3K9 methylation has been strongly correlated with DNA methylation (reviewed in [31]): Loss of the SUV39H1 and SUV39H2 H3K9 methyltransferases in mice simultaneously impairs accumulation of H3K9me3 across the genome [32] and accumulation of DNA methylation at pericentric major satellite repeats, but not at minor satellite or C-type retroviral repeats [13]. DNA methylation deficiencies were also noted in plants lacking H3K9 methyltransferases [12],[19] with one study reporting that maintenance of DNA methylation was affected [18]. To investigate the relationship between H3K9me3 and DNA methylation at *Rasgrf1*, we asked if H3K9me3 controlled by SUV39H1 and SUV39H2 affected imprinted DNA methylation at *Rasgrf1*. To address this, we performed methylation analysis on adult testes DNA using COBRA, bisulfite sequencing and a PCR assay that detected methylation status at a series of five *Hha*I sites in the DMD. Testes primarily contain cells of the germline, which will carry paternal epigenotypes, but some somatic cells are also present, which will carry both paternal and maternal epigenotypes. The COBRA analysis suggested that the DNA was hypomethylated in the SUV39H1- and SUV39H2-doubly deficient testes (Figure 7A). When we measured the extent of DNA methylation using *Hha*I site-spanning Q-PCR assays, it was clear that the loss of *Rasgrf1* DNA methylation was significant (Figure 7B). Bisulfite sequencing provided additional confirmation with higher resolution – there was a significant decrease in the number of DNA templates that were more than 80–100% methylated and an increase in the number that were 40–80% methylated in SUV39H1- and SUV39H2-doubly deficient testes (Figure 7C) but there was no change in the abundance of DNAs that were completely unmethylated. The reduction in DNAs with the 80–100% methylated paternal epigenotype, and the increase in DNAs with the 40–80% methylated epigenotype suggests that SUV39H1 and SUV39H2 control the efficiency with which imprinted DNA methylation is established in mice. In *Arabidopsis*, the SUVH4 H3K9 methyltransferase is known to control maintenance of DNA methylation [18].

## Discussion

We report here the epigenetic states that exist within a 12 kbp interval centered on the *Rasgrf1* ICR. Both parental alleles were marked by DNA methylation in somatic tissue on a 1.4 kbp segment at the very 5′ end of this 12,020 nt interval (D1–D2, Figure 2C, D). Downstream of this were segments that spanned the ICR that were paternally methylated in somatic DNA (D3–D8), and sperm DNA as well (D3–D5, Figure 2B,D). Not every CpG was assayed in this 12,020 interval, including those within the tandem repeats that constitute the DNA methylation programmer. H3K9me3 was present on both parental alleles at the core DMD immediately 5′ of the tandem repeats and within the ICR. H3K27me3 was present at this same location, but exclusively on the maternal allele. There was no appreciable dimethylation of these H3 residues at the core DMD (Figure 4A, B, C).

The tandem repeats, consisting of approximately 40 copies of a 41 nt unit, influenced the placement of histone and DNA methylation (Figures, 2B, D, 3 and 4D) and can be considered a *cis*-acting methylation programming sequence, one of only a few naturally occurring ones known in mammals. Paternal allele DNA methylation was particularly sensitive to these tandem repeats, which control establishment of DNA methylation in the male germline at a 400 nt core DMD lying just 5′ of the repeats (Figure 2B and [2]). The repeats also control spreading and maintenance of paternal allele DNA methylation in somatic tissue over a broader domain (Figure 2B, D and [3]).

Marking the core DMD with DNA methylation on the paternal allele and H3K27me3 on the maternal allele are coordinated and mutually exclusive events in wild type cells with DNA methylation largely confined to the core DMD on the paternal allele and H3K27me3 on the maternal allele (Figures 2D, 3B and 4). The mutual exclusion arises because one epigenetic mark antagonizes the placement of the other. Five independent lines of evidence led to this conclusion. First, MEFs taken from mice lacking DNA methylation on the paternal DMD inappropriately accumulated H3K27me3 on the paternal allele (Figure 4D). Second, MEFs treated with the DNA methyltransferase inhibitor, 5-azacytidine, accumulate elevated levels of H3K27me3 marks (Figure 5A). Third, MEFs taken from mice with a maternally transmitted *Rasgrf1* ICR transgene that lacked DNA methylation had H3K27me3 on the transgenic DMD, whereas H3K27me3 was excluded by manipulations that inappropriately placed DNA methylation on the transgene (Figure 5B, lower panel). Fourth, mutation of the *Suz12* component of PRC2, which is needed for activity of the EZH2 H3K27 methyltransferase in PRC2, ablated normal placement of H3K27me3 and enabled the maternal allele to inappropriately acquire DNA methylation (Figure 6A, B). Fifth, mutation of the *Yy1* gene, which is needed to recruit PRC2 to DNA and, like *Suz12*, is needed for effective placement of H3K27me3 also enabled the maternal allele to inappropriately acquire DNA methylation (Figure 6C). Other studies have documented the cross-dependency of some histone modifications and DNA methylation [11], [13], [14], [19], [33]–[37], and it has also been observed that H3K27me3 and DNA methylation can be mutually exclusive [21]. The studies described here provide evidence that H3K27me3 and DNA methylation are in fact mutually antagonizing epigenetic marks and that H3K27me3 facilitates allele-specific DNA methylation that exists at imprinted loci.

H3K9me3 was detected on both parental alleles indicating this mark is controlled differently from H3K27me3. However, it too participates in imprinted DNA methylation because the H3K9 methyltransferases, SUV39H1 and SUV39H2, are needed for optimal establishment of DNA methylation at the DMD in the male germline (Figure 7).

We do not know how DNA and H3K27 methylation antagonize each other's placement; however, the literature highlights several molecular and developmental events, as well as protein factors that may be involved. Among these is the transcriptional state that is known to influence which of two mutually exclusive histone modifications is placed by the competing activities of polycomb (PcG) and trithorax (Trx) group proteins [38]. Additionally, differentiation state is known to influence genome wide epigenetic patterns in ES, MEF and neuronal progenitor cells [39]. At *Rasgrf1*, developmental stage also influences epigenetic states [40]; the methylation programmer controls establishment of DNA methylation in the germline and maintenance in peri-implantation embryos [2],[3], but not later in development. Interestingly, this same period is a critical interval for control of H3K27 methylation [41]. Finally, there may be a role for CTCF in the mutual exclusion of H3K27 and DNA methylation at *Rasgrf1*. CTCF and its binding sites have been shown to influence *H19* DNA methylation [42]–[47] and CTCF binds at *Rasgrf1* as well [4]. Genome-wide ChIP analysis identified locations enriched for CTCF [48] and H3K27me3 [49] in MEFs and Chi squared analysis reveals a significant co-localization of these marks at imprinted versus non-imprinted loci (Table S3). This raises the possibility that, in addition to its role in preventing DNA methylation at other imprinted loci, CTCF helps to place H3K27me3 at *Rasgrf1*. CTCF functions in coordination with its binding partner, YY1, in activating the X chromosome [50] and YY1 also inhibits DNA methylation at *Rasgrf1* (Figure 6C), most likely through its ability to recruit PRC2 [22]–[24]. Depending upon the consensus sequence considered, between one and twelve YY1 sites are predicted to lie within the DMD and repeat region (data not shown). Like CTCF, YY1 sites are enriched at other imprinted loci as well [51]. CTCF has additional binding partners including CHD8, which is associated with DNA methylation [52]. Using ChIP analysis, we could not detect CHD8 on either *Rasgrf1* allele (data not shown), suggesting that at *Rasgrf1*, other CTCF binding partners and functions might be more important, such as YY1.

Normal placement of DNA methylation on the paternal allele and H3K27me3 on the maternal allele both require the same tandemly repeated DNA sequence element, which we previously showed has DNA methylation programming activity ([2],[3] and YJP, HH and PDS unpublished). However, DNA methylation is more rigidly dependent on the repeated sequence than are the histone modifications. Whereas DNA methylation on the paternal core DMD was typically completely lost when the repeats were deleted, H3K27me3 and H3K9me3 on the maternal DMD were respectively reduced to levels only 1/2 and 1/6 of those seen when the repeats were present. Repeated sequences have been shown to have methylation programming activity in other systems [53],[54]. Notably, at the *DM1* locus in humans, a repetitive element is associated with heterochromatin accumulation [55]. Interestingly, like the maternal *Rasgrf1* DMD and repeat sequences [4], the *DM1* repeat also is a CTCF-binding insulator. CTCF appears to restrict the boundary of heterochromatinization at *DM1*, but it is not known if CTCF has a similar effect at *Rasgrf1*. Sequences with appreciable similarity to the *Rasgrf1* tandem repeats are not abundant in the mouse genome. However, the *Rasgrf1* repeat unit has striking similarity to the B repeat sequences on *Xist* (Figure S4). Because *Xist* RNA regulates placement of H3K27me3 on the inactive X chromosome and at an autosomal transgenic site in *cis* [56],[57], it is possible there is mechanistic overlap between epigenetic regulation by *Xist* and the *Rasgrf1* repeats.

We do not know what functional motifs enable the methylation programmer at *Rasgrf1* to control either DNA or H3 methylation. Its repeated nature may be sufficient [54], possibly involving an RNA-dependent mechanism [58]. Other potentially important features include the CpG present in 36 of the 40 repeat units; GGGG tetramers that may facilitate the formation of G-quadruplex structures [59], which in turn may alter the sensitivity of DNA to methyltransferase action [60]; or CTCF sites known to lie in the *Rasgrf1* methylation programmer [4]. BORIS, the male germline paralog of CTCF [61], may also be important for function of the *Rasgrf1* methylation programmer.

**Figure 8:**
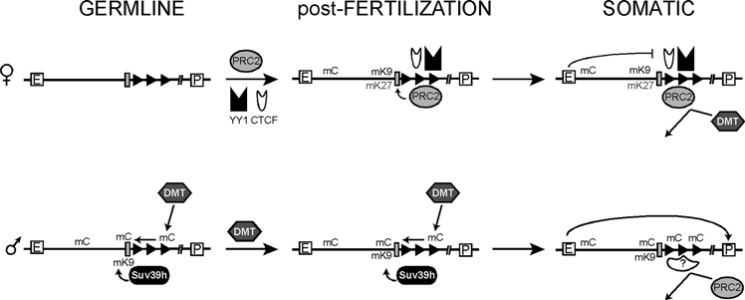
Model summarizing epigenetic control at the ICR of *Rasgrf1*. The maternal allele (top) recruits YY1, PRC2 components, and possibly CTCF early during development or gametogenesis. H3K27 and H3K9 are both methylated (mK27 and mK9 respectively) in the vicinity of the core DMD (grey box), with optimal methylation depending upon the tandem repeats (rightward pointing black triangles, which constitute the methylation programmer). Once placed, H3K27me3 can exclude DNA methylation. On the paternal allele (bottom), the methylation programmer is active in the germline where it directs DNA methylation to the DMD [2] by a process optimized by SUV39H1 and SUV39H2 directed H3K9 methylation, and in the pre-implantation embryo where it maintains it [3]. Once established in the germline, DNA methylation on the core DMD can expand to surrounding sequences, and exclude subsequent H3K27me3 during somatic development. In the neonatal brain, where *Rasgrf1* shows imprinted expression, recruitment of CTCF to the maternal allele allows the enhancer blocking activity of the DMD to silence the maternal allele by restricting interactions between a putative upstream enhancer (E) and the downstream promoter (P), while exclusion of CTCF by methylated DNA at the paternal allele allows expression [4].

Figure 8 describes a model for the placement of DNA methylation and H3K27me3 in response to the *Rasgrf1* methylation programmer, their antagonism, and the developmental timing of these events. However, it is unlikely that a universal rule dictates the regulation of DNA and H3K27 methylation at all loci within a species or among species. In human cell lines, some loci have been found at which DNA and H3K27 methylation occur simultaneously with one mark requiring the placement of the other [62], whereas in *Arabidopsis*, DNA methylation does not seem to be closely associated with H3K27me3 [20] and in fact can be mutually exclusive [21]. Nonetheless, identifying the various rules that influence epigenetic programming of normal developmental states will provide insights for manipulating them for therapeutic benefit.

## Materials and Methods

### Mouse Strains and Mutants

Mice used for DNA methylation analysis across the 12 kbp interval were F1 progeny of PWK and 129S4SvJae parents. Polymorphisms in these strains facilitated the assignment of a given clone from bisulfite PCR to one of the two parental alleles. Mice used to prepare MEFs for ChIP analysis across the 12 kbp interval were from strain 129S4SvJae backcrossed to C57BL/6 and included wild type animals, animals carrying a repeat deletion [2] and animals containing an engineered polymorphism at the DMD that did not disrupt imprinting [3]. All allele specific ChIP analyses were done using MEFs from mice carrying one of these mutations. Mice carrying the *Rasgrf1* ICR transgene will be described in a separate report (YJP, HH, PDS, in preparation). Previous reports describe the *Suz12* mutation and preparation of homozygous ES cells and embryoid bodies [26] and the *Yy1* mutation and preparation of trophoblast outgrowths [27].

### Chromatin Immunoprecipitation (ChIP) Analysis

MEFs from 13.5 day old F1 embryos of C57BL/6 and 129S4Jae parents were used for ChIP analysis as described in Text S1. Modified histone-specific antibodies were from Millipore/Upstate (H3K9me2 item 07-441 lot 29698, H3K9me3 item 07-442 lot 24416, H3K27me2 item 07-452 lot 24461, H3K27me3 item 07-449 lot 24440) and Thomas Jenuwein, IMP, Austria (H3K9me3) [15]. Specificity of antibody from Thomas Jenuwein's lab has been reported [15]. Validations of commercial antibody specificities are publicly available from the manufacturer (see http://www.millipore.com for certificates of analysis for each catalog item and lot number). The DNA recovered after ChIP was used for Q-PCR with input chromatin and mock immunoprecipitations without antibody serving as controls. Q-PCR was performed in triplicate with SYBR green detection using primers listed in Table S1. Ratios of bound to input signals are reported.

### DNA Methylation Analysis

Treatment of genomic DNA with bisulfite was performed as previously described [2], with the added difference that we used 1.5 M betaine and 5% DMSO to enhance the yield in PCR of AT-rich, converted DNA. ExTaq HS DNA polymerase (Takara, Japan) was used for hotstart PCR. Primers used are provided in Table S1 and additional experimental details are in Text S1. The bisulfite converted and amplified DNA was either cloned and sequenced or subjected to COBRA [28] using *Bst*UI digestions. In this assay, cytosine methylation enables digestion, whereas absence of methylation prevents it. Assays for DNA methylation using *Hha*I digested DNAs were described [2].

## Supporting Information

Figure S1 CpG dinucleotides and methylated DNA centered at the DMD. A,B,C. The distribution of A, T, C and G over the 220 kb cluster show that there is a predominant accumulation of cytosines over the DMD and repeat region. The DMD repeat region has large amount of C and G together. There are two CpG islands in the region: one, which is the DMD and the other, which is in the promoter region of Rasgrf1 (bottom panel). D. Southern blot analysis of the two CpG islands using methylation sensitive restriction enzymes and tail DNA show that there is monoallelic methylation at the DMD (left panel) but no methylation of the promoter region CpG island (right panel). P (PstNI), N (NotI, methylation sensitive), E (EcoRI), Br (BsrBI, methylation sensitive). Bands diagnostic for the methylated (+) and unmethylated (−) states are indicated.
(0.55 MB TIF)

Figure S2 Specificity controls for antibodies used in ChIP. Representative gel analysis of ChIP results indicating the specificity of the antibodies for the histone modification analysis in this study. Antibodies specific to H3K9me2, H3K9me3, H3K27me2, and H3K27me3 show enrichment for H3K9me3 and H3K27me3 at the DMD. Positive control PCRs for H3K9me2 and H3K27me2 (Charlie [1]), H3K9me3 (Actin), and H3K27me3 (Hoxa9 [2]) are included as well as a test for the Rasgrf1 DMD. NTC, no template control; WCE, whole cell extract not immunoprecipitated; no ab, mock precipitations done without antibody.
(0.12 MB TIF)

Figure S3 Mutual exclusion of H3K27 and DNA methylation. H3K27 and DNA methylation data from figures 2 and 3 were redrawn to highlight the mutual exclusion of H3K27me3 and DNA methylation. (A) Modifications present at *Rasgrf1* in wild type MEFs show that paternal DNA methylation (green) is largely even over the region, while maternal DNA methylation (red) is absent over the DMD but present upstream and downstream. Strikingly, H3K9me3 and H3K27me3 are perfectly confined to the DMD. (B) Modifications in the paternal allele in +/RepΔ mice. DNA methylation (black) is lost from the DMD and downstream, allowing encroachment of H3K27me3 into these regions (red).
(0.15 MB TIF)

Figure S4 Dot plot of Xist and the *Rasgrf1* ICR. (A) Xist sequences, including the A, B, C, D and E repeats (17 kb) and *Rasgrf1* sequences including the DMD and repeats (5 kb) were aligned in a dot plot matrix. (B) Detail of the dot plot matrix in A that includes the Xist B element and the *Rasgrf1* repeats.
(0.24 MB TIF)

Table S1 Primers used for PCR amplification.
(0.06 MB DOC)

Table S2 Clones sequenced for analysis in Figure 2. DNAs from neonatal brains, taken from mice with the three indicated genotypes, and from sperm with the two indicated genotypes, were subjected to bisulfite PCR and the PCR products were cloned and sequenced. Primers used to amplify regions D1 through D8 are listed in Table S1. This table reports the number of clones sequenced that correspond to the maternal and paternal alleles for brain DNA. Assignment of individual clones to the maternal or paternal allele required the use of polymorphisms between PWK and 129S4Jae parents of F1 DNAs, as described in Supporting Methods. Note that no polymorphisms were present in D3 and D6 so allele specific methylation was not determined (nd) in neonatal brain DNA from F1 mice in those regions.
(0.08 MB DOC)

Table S3 Enhanced colocalization of CTCF and H3K27me3 at imprinted loci. Whole genome H3K27me3 ChIP data for imprinted and known genes in MEF cells were downloaded from http://www.broad.mit.edu/seq_platform/chip/ and experimentally verified CTCF site data were downloaded from http://insulatordb.utmem.edu/browse.php. After filtering the H3K27me3 ChIP data for sites with a read score of two or higher, the data sets were added as custom tracks on the UCSC Genome Browser and intersected in the intervals spanning 17,553 known genes and 53 imprinted gene regions. The intervals examined included the 100 kb 5′ of each gene (+100), sequences between the 5′ and 3′ ends of the genes (G), 100 kb 3′ of the genes (−100), and the entire stretch from 100 kb 5′ to 100 kb 3′ of each gene region (+100 to 100). The number of times H3K27me3 colocalized with CTCF in the indicated intervals is reported. The frequency of colocalization per kb was calculated for each interval examined, and the values for each of the known gene intervals were used to calculate an expected value for the corresponding imprinted gene intervals, given the total number of kbp in each of the imprinted gene intervals examined. The number of observed and expected colocalizations in the imprinted intervals was then used in Chi-square analysis.
(0.06 MB DOC)

Text S1 Supporting methods and references.
(0.04 MB DOC)
