# Supplementary figures and images for: Antagonism between DNA and H3K27 Methylation at the Imprinted Rasgrf1 Locus
Source: PLoS Genet. 2008 Aug 1;4(8):e1000145. doi: 10.1371/journal.pgen.1000145 (PMC2475503; doi:10.1371/journal.pgen.1000145)

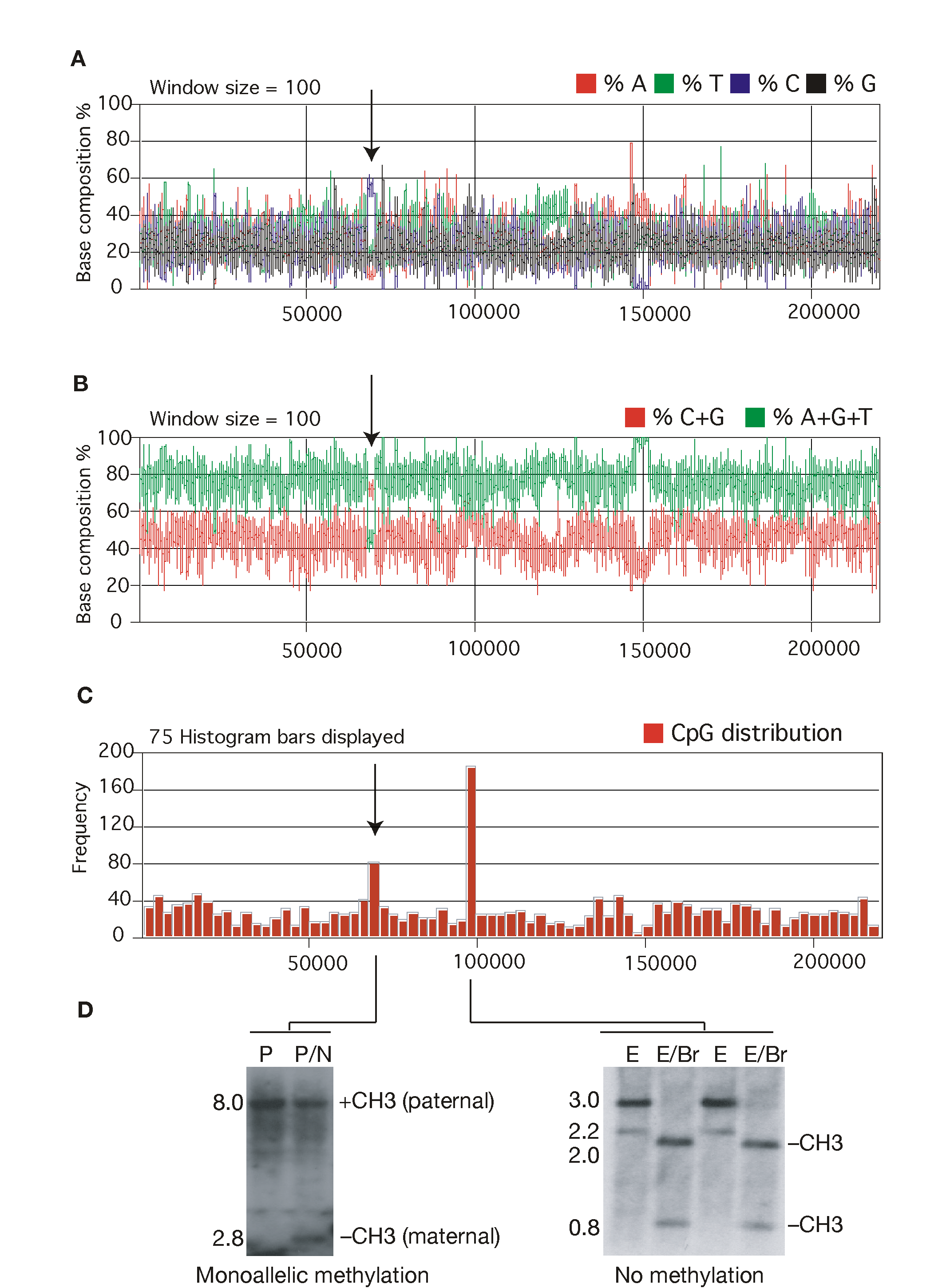

Supplement: Figure S1 — CpG dinucleotides and methylated DNA centered at the DMD. A,B,C. The distribution of A, T, C and G over the 220 kb cluster show that there is a predominant accumulation of cytosines over the DMD and repeat region. The DMD repeat region has large amount of C and G together. There are two CpG islands in the region: one, which is the DMD and the other, which is in the promoter region of Rasgrf1 (bottom panel). D. Southern blot analysis of the two CpG islands using methylation sensitive restriction enzymes and tail DNA show that there is monoallelic methylation at the DMD (left panel) but no methylation of the promoter region CpG island (right panel). P (PstNI), N (NotI, methylation sensitive), E (EcoRI), Br (BsrBI, methylation sensitive). Bands diagnostic for the methylated (+) and unmethylated (−) states are indicated. (0.55 MB TIF) [file pgen.1000145.s001.tif]

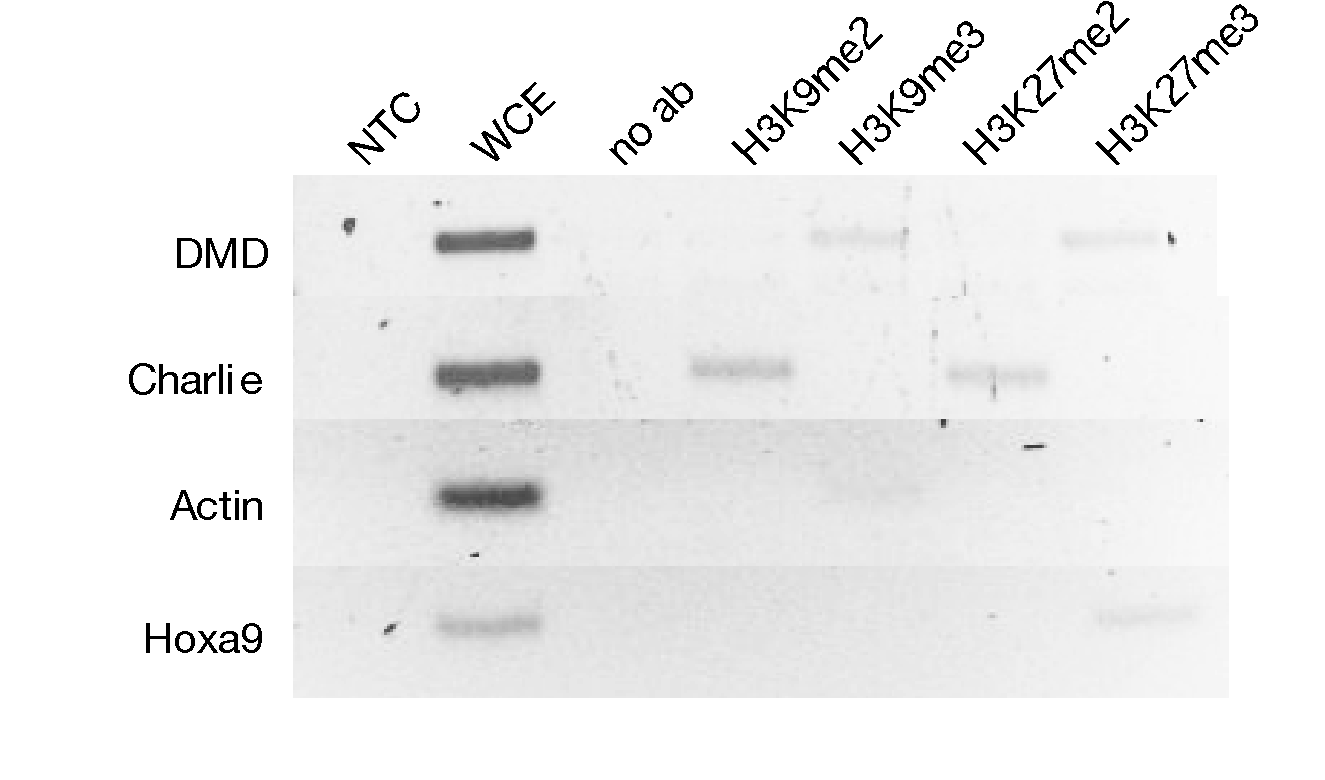

Supplement: Figure S2 — Specificity controls for antibodies used in ChIP. Representative gel analysis of ChIP results indicating the specificity of the antibodies for the histone modification analysis in this study. Antibodies specific to H3K9me2, H3K9me3, H3K27me2, and H3K27me3 show enrichment for H3K9me3 and H3K27me3 at the DMD. Positive control PCRs for H3K9me2 and H3K27me2 (Charlie [1]), H3K9me3 (Actin), and H3K27me3 (Hoxa9 [2]) are included as well as a test for the Rasgrf1 DMD. NTC, no template control; WCE, whole cell extract not immunoprecipitated; no ab, mock precipitations done without antibody. (0.12 MB TIF) [file pgen.1000145.s002.tif]

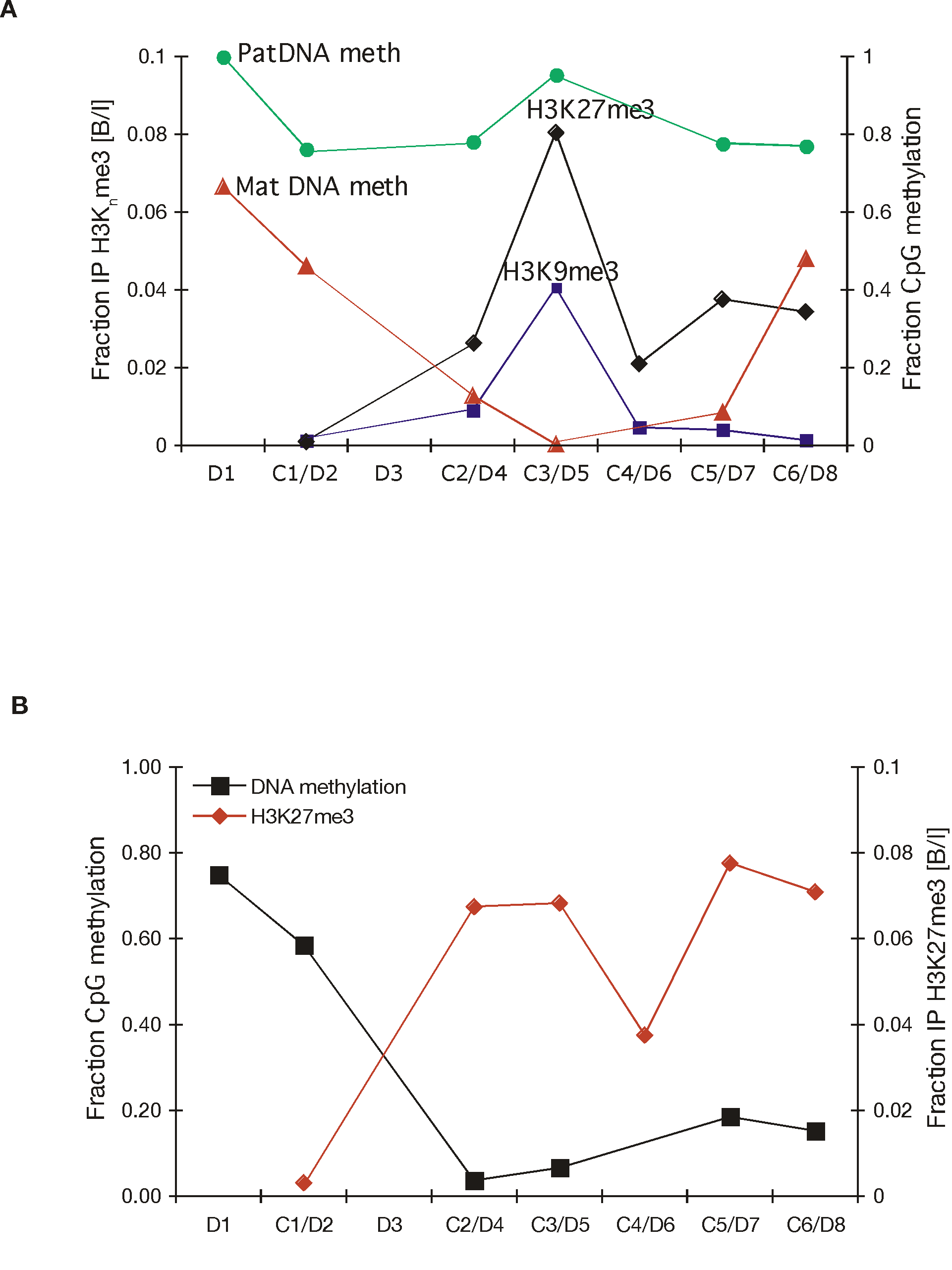

Supplement: Figure S3 — Mutual exclusion of H3K27 and DNA methylation. H3K27 and DNA methylation data from figures 2 and 3 were redrawn to highlight the mutual exclusion of H3K27me3 and DNA methylation. (A) Modifications present at Rasgrf1 in wild type MEFs show that paternal DNA methylation (green) is largely even over the region, while maternal DNA methylation (red) is absent over the DMD but present upstream and downstream. Strikingly, H3K9me3 and H3K27me3 are perfectly confined to the DMD. (B) Modifications in the paternal allele in +/RepΔ mice. DNA methylation (black) is lost from the DMD and downstream, allowing encroachment of H3K27me3 into these regions (red). (0.15 MB TIF) [file pgen.1000145.s003.tif]

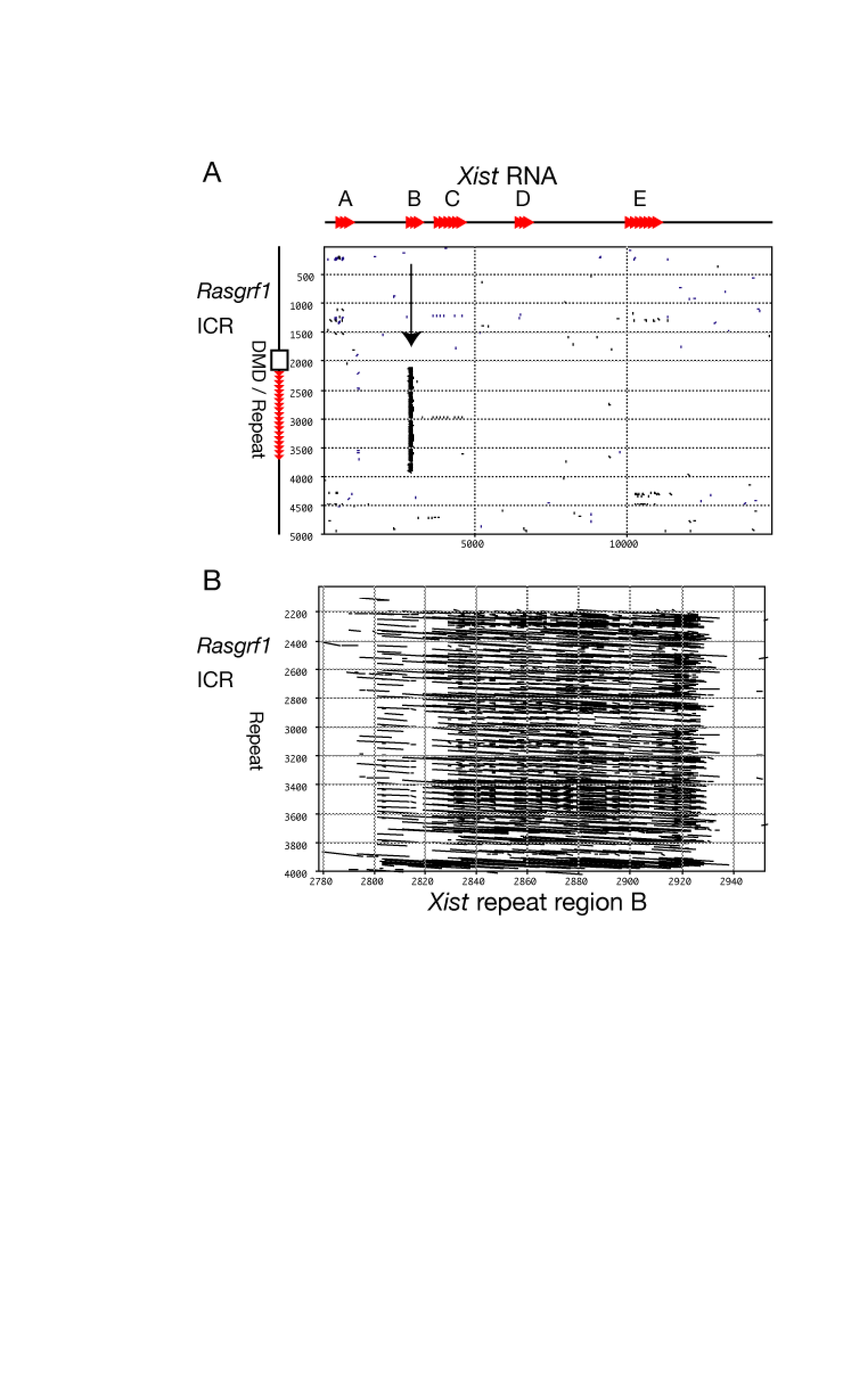

Supplement: Figure S4 — Dot plot of Xist and the Rasgrf1 ICR. (A) Xist sequences, including the A, B, C, D and E repeats (17 kb) and Rasgrf1 sequences including the DMD and repeats (5 kb) were aligned in a dot plot matrix. (B) Detail of the dot plot matrix in A that includes the Xist B element and the Rasgrf1 repeats. (0.24 MB TIF) [file pgen.1000145.s004.tif]
