# Supplementary material for: Antagonism between DNA and H3K27 Methylation at the Imprinted Rasgrf1 Locus
Source: PLoS Genet. 2008 Aug 1;4(8):e1000145. doi: 10.1371/journal.pgen.1000145 (PMC2475503; doi:10.1371/journal.pgen.1000145)
Supplement: Table S1 — Primers used for PCR amplification. (0.06 MB DOC) [file pgen.1000145.s005.doc]

#### Table S1. Primers used for PCR amplification.

| **DNA Amplified** | **Primer Pair** | **Lab code** |
| --- | --- | --- |
| *Rasgrf1*^tm1Pds^  Repeat deletion | 5'-CACTTCGCTACCGTTTCGC-3'  5'-TGTCCTCCACCCCTCCACC-3' | P3 PDS16  P4 PDS17 |
| *C1* | 5’-GCCCTCCCTGTCTGTATGAG-3’  5’-TGTCAAACCCCCAAACTAGC-3’ | PDS104  PDS105 |
| *C2* | 5’-ATACGTGCACACAGGCAAAA-3’  5’-GGCCTGGCATAGTAGCAGAA-3’ | PDS650  PDS651 |
| *C3 (Core DMD)* | 5'-CACATCCATCCGTGGCTACCGCTATTGCTGT-3'  5'-GCGAAGTGCGGCAGCAGCAGCGA-3' | P1 PDS12  P2 PDS13 |
| *C4* | 5’-GGGTGGGTCTTTCTTTGTCA-3’  5’-CAACCGATAAGCCAAGTAGGA-3’ | PDS648  PDS649 |
| *C5* | 5’-AACAATTCTGGCTCCCACAG-3’  5’-CCTCCCTCCAAAAGGACACT-3’ | PDS108  PDS109 |
| *C6* | 5’-TAGTGATCCCCTTGCCTTTG-3’  5’-CCATATCACACCCTGGCTCT-3’ | PDS106  PDS107 |
| *D1* | 5’- TGTTGTGTTATGGTTTATATATGGAGGTTAGAG-3’  5’-ACACCTAAAACCCATACAACTATTTCCCTAATA-3’ | PDS629-Y  PDS630 |
| *D2* | 5’-TTAGTGATTGTGATGATTTTTTGTTTGAGTT-3’  5’-TTAAAATTCTATCAAACCCCCAAACTAACTAC-3’ | PDS368  PDS369 |
| *D3* | 5’-AGTGTATTGTGTTTTTATTGGTTATTTTAAAGGATAGAAT-3’  5’-AAACCATCACAAAAAACCACACAACTC-3’ | PDS559  PDS356 |
| *D4* | 5’-GGGATTTAAAATGTTTTTTTTTGGTTATTAGGGAT-3’  5’-ACATTCTCAACAAAAACAATAACCTACCTA-3’ | PDS269  PDS270 |
| *D5* | 5’-GGAATTTTGGGGATTTTTTAGAGAGTTTATAAAGT-3’  5’-CAAAAACAACAATAATAACAAAAACAAAAACAATAT-3’ | PDS271  PDS272 |
| *D5 (RepΔ)* | 5’-GGAATTTTGGGGATTTTTTAGAGAGTTTATAAAGT-3’  5’-CTATATTAAATCCTTTTATCCACTATCCTCCACCC-3’ | PDS271  PDS287 |
| *D6* | 5’-TAGTTGGAGATATTTTGATGAGGAAGATTAGATTTG-3’  5’-AACCATCCTAATTAACAAAACAAAACCC-3’ | PDS623  PDS4 |
| *D7* | 5’-AAGGTATGTGAATTTATATGTGGTTGGGAA-3’  5’-TCCATTCCTCCCTCCAAAAAAACAC-3’ | PDS563  PDS564 |
| *D8* | 5’-TGGGAGGAAGGATTGTGTATATATGGAT-3’  5’-ACTTCCAAAACACTCTCTCTACTTTCTCTA-3’ | PDS275  PDS276 |
| *Rpl32* | 5’-CATGCACACAAGCCATCTACTCA-3’  5’-TGCTCACAATGTGTCCTCTAAGAAC-3’ | PDS72  PDS73 |
| *Actin* | 5'-CAGTTCGCCATGGATGACGATATCG-3'  5'-CCGCGAAGCCGGCTTTGCACATG-3' | PDS38  PDS39 |
| *Transgenic DMD* | 5’-CACATCCATCCGTGGCTACCGCTATTGCTGT-3’  5’-CCTGCAGGTCGACATAACTTC-3’ | pYJC6F2  pYJC6R2 |
| *Charlie* | 5’—TTGAGAATCGGATGGGAGAC-3’  5’-AAGAACTGTCTTATTCAGGC-3’ | PDS660  PDS661 |
| *Hoxa9* | 5’-ACCGACTCTGCCAGCTTTAC-3’  5’-TCTCCCTTCTCAAACCCTCA-3’ | PDS646  PDS647 |
| *COBRA Primers* | 5’-AGAGAGTATGTAAAGTTAGAGTTGTGTTGTTG-3’  5’-CAAAAACAACAATAATAACAAAAACAAAAACAATAT-3 | PDS225  PDS272 |
