# Supplementary material for: Antagonism between DNA and H3K27 Methylation at the Imprinted Rasgrf1 Locus
Source: PLoS Genet. 2008 Aug 1;4(8):e1000145. doi: 10.1371/journal.pgen.1000145 (PMC2475503; doi:10.1371/journal.pgen.1000145)
Supplement: Table S2 — Clones sequenced for analysis in Figure 2. DNAs from neonatal brains, taken from mice with the three indicated genotypes, and from sperm with the two indicated genotypes, were subjected to bisulfite PCR and the PCR products were cloned and sequenced. Primers used to amplify regions D1 through D8 are listed in Table S1. This table reports the number of clones sequenced that correspond to the maternal and paternal alleles for brain DNA. Assignment of individual clones to the maternal or paternal allele required the use of polymorphisms between PWK and 129S4Jae parents of F1 DNAs, as described in Supporting Methods. Note that no polymorphisms were present in D3 and D6 so allele specific methylation was not determined (nd) in neonatal brain DNA from F1 mice in those regions. (0.08 MB DOC) [file pgen.1000145.s006.doc]

#### Table S2. Clones sequenced for analysis in Figure 2.

|  |  | D1 | D2 | D3 | D4 | D5 | D6 | D7 | D8 | TOTALS |
| --- | --- | --- | --- | --- | --- | --- | --- | --- | --- | --- |
| NEONATAL BRAIN |  |  |  |  |  |  |  |  |  |  |
|  |  |  |  |  |  |  |  |  |  |  |
| +/+ | Maternal | 3 | 20 | nd | 16 | 11 | nd | 32 | 17 | 99 |
|  | Paternal | 3 | 13 | nd | 16 | 9 | nd | 13 | 17 | 71 |
|  |  |  |  |  |  |  |  |  |  |  |
| +/RepΔ | Maternal | 3 | 2 | nd | 9 | 6 | nd | 8 | 17 | 45 |
|  | Paternal | 4 | 18 | nd | 9 | 8 | nd | 7 | 9 | 55 |
|  |  |  |  |  |  |  |  |  |  |  |
| RepΔ/+ | Maternal | 2 | 13 | nd | 7 | 10 | nd | 18 | 18 | 68 |
|  | Paternal | 4 | 6 | nd | 10 | 7 | nd | 5 | 7 | 39 |
|  |  |  |  |  |  |  |  |  |  |  |
| SPERM |  |  |  |  |  |  |  |  |  |  |
|  |  |  |  |  |  |  |  |  |  |  |
| +/+ |  | 7 | 7 | 9 | 7 | 10 | 8 | 5 | 7 | 52 |
| RepΔ/RepΔ |  | 8 | 7 | 10 | 16 | 9 | 11 | 8 | 13 | 71 |
|  |  |  |  |  |  |  |  |  |  |  |
| TOTALS |  | 34 | 86 | 19 | 90 | 70 | 19 | 96 | 105 | 500 |
