# Supplementary material for: Antagonism between DNA and H3K27 Methylation at the Imprinted Rasgrf1 Locus
Source: PLoS Genet. 2008 Aug 1;4(8):e1000145. doi: 10.1371/journal.pgen.1000145 (PMC2475503; doi:10.1371/journal.pgen.1000145)
Supplement: Table S3 — Enhanced colocalization of CTCF and H3K27me3 at imprinted loci. Whole genome H3K27me3 ChIP data for imprinted and known genes in MEF cells were downloaded from http://www.broad.mit.edu/seq_platform/chip/ and experimentally verified CTCF site data were downloaded from http://insulatordb.utmem.edu/browse.php. After filtering the H3K27me3 ChIP data for sites with a read score of two or higher, the data sets were added as custom tracks on the UCSC Genome Browser and intersected in the intervals spanning 17,553 known genes and 53 imprinted gene regions. The intervals examined included the 100 kb 5′ of each gene (+100), sequences between the 5′ and 3′ ends of the genes (G), 100 kb 3′ of the genes (−100), and the entire stretch from 100 kb 5′ to 100 kb 3′ of each gene region (+100 to 100). The number of times H3K27me3 colocalized with CTCF in the indicated intervals is reported. The frequency of colocalization per kb was calculated for each interval examined, and the values for each of the known gene intervals were used to calculate an expected value for the corresponding imprinted gene intervals, given the total number of kbp in each of the imprinted gene intervals examined. The number of observed and expected colocalizations in the imprinted intervals was then used in Chi-square analysis. (0.06 MB DOC) [file pgen.1000145.s007.doc]

#### Table S3. Enhanced colocalization of CTCF and H3K27me3 at imprinted loci

| **Genes** | **Number of times H3K27me3 and CTCF colocalize**  **in MEFs** | **Interval** | **Number of Kbp**  **in interval** | **CTCF and H3K27me3 colocalized sites per kbp** | **Expected CTCF and H3K27me3 colocalized sites per imprinted gene** | **Chi square** | **P value** |
| --- | --- | --- | --- | --- | --- | --- | --- |
| Known | 60 | +100 | 654506 | 9.17E-05 | - | - | - |
| 17,553 | 23 | G | 840861 | 2.74E-05 | - | - | - |
|  | 118 | -100 | 1068023 | 1.10E-04 | - | - | - |
|  | 201 | +100 to -100 | 2563390 | 7.84E-05 | - | - | - |
| Imprinted | 8 | +100 | 6997 | - | 6.41E-01 | 8.44E+01 | 4.01E-20 |
| 53 | 1 | G | 2981 | - | 8.15E-02 | 1.03E+01 | 1.30E-03 |
|  | 12 | -100 | 4195 | - | 4.64E-01 | 2.87E+02 | 2.09E-64 |
|  | 21 | +100 to -100 | 14173 | - | 1.11E+00 | 3.56E+02 | 2.18E-79 |
