# Supplementary material for: Antagonism between DNA and H3K27 Methylation at the Imprinted Rasgrf1 Locus
Source: PLoS Genet. 2008 Aug 1;4(8):e1000145. doi: 10.1371/journal.pgen.1000145 (PMC2475503; doi:10.1371/journal.pgen.1000145)
Supplement: Text S1 — Supporting methods and references. (0.04 MB DOC) [file pgen.1000145.s008.doc]

### SUPPORTING METHODS

#### Bisulfite assay details

On the day of bisulfite treatment, we prepared fresh 10M NaOH, 6.3M NaOH, 20mM hydroquinone (Sigma H9003) and 3.9M bisulfite solution. The bisulfite solution included 16.2g of Na bisulfite in 30ml dH2O, pH adjusted to 5.1 with 10M NaOH. Then we added 1.32ml of 20mM hydroquinone and adjusted the total volume into 40ml, sterilized the solution with a 0.2μm filter and stored protected from light. We used 2μg of genomic DNA, sheared by passage through a 30g needle 15 times, and purified by phenol/chloroform extraction, followed by alcohol precipitation. We resuspended DNA in 20μl dH2O and transferred to a 0.65ml tube, heated at 95°C for 5min in PCR machine and quenched on ice. Then we added 1μl of 6.3M NaOH, incubated at 39°C for 30min, followed by 208μl bisulfite solution mixed and incubated the reaction at 55°C for 16hr in the dark in a PCR machine, with heating to 95°C for 5min every 3 hours. At the end of the incubation, we desalted samples using a Qiagen Quickspin column, following the manufacturers instructions, and eluted the sample in 50μl EB (Qiagen). We completed the reaction by adding 2.5μl of 6.3M NaOH (final 0.3M) and incubated at 37°C for 15 min. Finally, we purified DNA samples using Qiagen Quickspin columns, after adjusting the pH to 5 using 3M Na acetate, and eluted DNA with 30μl of warm EB, allowing the EB to stand in the column for 1-2 min. We used 2μl DNA for PCR and cloned the products into TOPO pCR2.1 (Invitrogen) using the manufacturers instructions. Primers used for PCR of bisulfite treated DNAs are listed in Supporting Table S1. Note for COBRA analysis of P9 EB from materials carrying *Suz12* and *Yy1* mutations, we used a semi-nested PCR assay for region D5 (see Fig. 1) with primers 225/272 in the first round and 271/272 in the second round.

#### Assigning bisulfite sequences to their parent of origin

In order to determine the DNA methylation state using bisulfite sequencing shown in Fig. 2, we used F1 progeny of PWK and 129S4Jae parents. We sequenced DNA from the inbred parents in the vicinity of regions D1-D8 and identified indels and SNPs in regions D1, D2, D4, D5, D7 and D8. When analyzing a given bisulfite sequence from the F1 DNA, we could readily determine the parent of origin by determining which indels or SNPs were present. Note that C⇔ T polymorphisms were uninformative because bisulfite treatment creates T residues from C residues in the PCR product.

#### ChIP assay details

MEF cells no older than passage four were grown in to confluence in DMEM with 10% fetal calf serum on 15 cm dishes. We cross linked chromatin with formaldehyde by adding 675μl of a 37% solution (1% final concentration) directly to 25 ml of culture medium and rotating cells lightly at room temperature for 10 min. The cross linking was quenched by adding 1 ml 2.5 M glycine (final 100 mM) and incubating for an additional 10 min. Cells were then rinsed with phosphate buffered saline containing proteinase inhibitor mix (PBS complete), used according to the manufacturer’s instructions (Roche, #11697498001). Cells were then scraped into 2 ml PBS complete, centrifuged at 4000xg for 5 minutes, and washed again with PBS complete. Cells were resuspended in lysis buffer (1% SDS, 10mM EDTA and 50mM Tris, pH 8.1 supplemented with proteinase inhibitors) to a concentration of 1-1.5x10^7^ cells /ml and frozen in 1ml-aliquots at –80°C for future use. For immunoprecipitations, we thawed frozen cells, added fresh proteinase inhibitors, supplemented with Pepstatin A (0.7μg/ml) and sonicated samples on ice until the DNA was sheared to ~500 bp. Chromatin was then diluted to 10 mls in dilution buffer (0.01% SDS, 1.1% Triton X- 100, 1.2mM EDTA, 16.7mM Tris-HCl, pH 8.1, 167mM NaCl supplemented with proteinase inhibitors). Two ml aliquots were used for each precipitation and for no antibody and input controls. Lysates were precleared with 60μl protein-G-agarose beads coated with SS-DNA and BSA (Millipore #16-201), for 1hr at 4°C followed by centrifugation at 2500xg, 1 min. Supernatants were transferred to new tube and desired antibody added (5-10μl from a 100μg/ml stock) and samples were rotated over night at 4°C. The next day, protein-G-agarose beads were added to samples (except input controls) which were rotated for 2h at 4 °C followed by centrifugation at 2500xg, 1 min. After discarding supernatant, we washed the beads twice in low salt buffer (0.1% SDS, 1% Triton X-100, 2mM EDTA, 20mM Tris-HCl, pH 8.1, 150mM NaCl), twice in high salt buffer (0.1% SDS, 1% Triton X-100, 2mM EDTA, 20mM Tris-HCl, pH 8.1, 500mM NaCl) once in LiCl buffer (0.25M LiCl, 1% IGEPAL CA630, 1% sodium deoxycholate, 1mM EDTA, 10mM Tris, pH 8.1) and twice in TE (10mM Tris pH 8.8, 1mM EDTA). Each wash was for 5 minutes with rotation at 4°C. Chromatin was eluted from beads using 200μl elution buffer (1% SDS, 0.1M NaHCO_3_) twice at 65°C and we pooled the eluted materials. We then reversed the cross links by adding 16μl 5M NaCl and incubating samples at 65°C for 4-6 h. We did this as well with material reserved for the input control. Protein was removed by adding 20μl 1M Tris (pH6.5), 10µl 0.5 M EDTA, and 1-2μl 10mg/ml Proteinase K followed by a 1h incubation at 45°C and an extraction with phenol/chloroform. DNA was transferred to new tubes containing 2-5μl Glycoblue (Ambion, #9516) and precipitated by adding 2 volumes ethanol. We ran Q-PCR on DNA after dissolving it in 50μl 10 mM Tris, pH 8.5.

Modified histone-specific antibodies were from Millipore/Upstate (H3K9me2 item 07-441 lot 29698, H3K9me3 item 07-442 lot 24416, H3K27me2 item 07-452 lot 24461, H3K27me3 item 07-449 lot 24440) and Thomas Jenuwein, IMP, Austria (H3K9me3) [1]. Specificity of antibody from Thomas Jenuwein’s lab has been reported [1]. Validations of commercial antibody specificities are publicly available from the manufacturer (see [http://www.millipore.com](http://www.millipore.com/) for certificates of analysis for each catalog item and lot number). These used a combination of methods including immunoblots of membranes containing an array of synthetic methylated histone peptides and solution binding assays. Additionally, immunoblots of membranes containing mammalian histones were used in competition tests that assayed the ability of immunogens and related peptides with distinct sites or numbers of methyl modifications to compete for antibody binding. Anti H3K27me3 binding to mammalian histones was modestly competed by a peptide containing dimethylated K27, however, no H3K27me2 was detected at *Rasgrf1* (Fig. 4) indicating any recognition of H3K27me2 by antibody designed to recognize H3K27me3 did not confound our assays at *Rasgrf1*. Note that in our hands the commercial antibodies produced similar results as those obtained by others using independently prepared noncommercial antibodies with the same specificities (Supporting Fig. S2).

### SUPPORTING REFERENCES

1. Martens JH, O'Sullivan RJ, Braunschweig U, Opravil S, Radolf M, et al. (2005) The profile of repeat-associated histone lysine methylation states in the mouse epigenome. Embo J 24: 800-812.

2. Schlesinger Y, Straussman R, Keshet I, Farkash S, Hecht M, et al. (2007) Polycomb-mediated methylation on Lys27 of histone H3 pre-marks genes for de novo methylation in cancer. Nat Genet 39: 232-236.

3. Peters AH, Kubicek S, Mechtler K, O'Sullivan RJ, Derijck AA, et al. (2003) Partitioning and plasticity of repressive histone methylation states in mammalian chromatin. Mol Cell 12: 1577-1589.
